# Widespread rescue of Y-linked genes by gene movement to autosomes

**DOI:** 10.1186/s13059-015-0686-1

**Published:** 2015-06-09

**Authors:** John H. Malone

**Affiliations:** Institute of Systems Genomics, Department of Molecular and Cell Biology, University of Connecticut, Storrs, Connecticut 06269 USA

## Abstract

A new study provides evidence that gene transposition from sex chromosomes to autosomes is a conserved phenomenon across mammalian species that rescues dosage-sensitive genes.

## Introduction

Theoretical models and empirical data suggest that sex chromosomes evolve from autosomes through the acquisition of a sex determination gene. As population genetic forces act on sex chromosomes, genes are lost from the Y chromosome, thus transforming the content of the sex chromosome gene pairs from a balanced state of two copies to an unbalanced state of one copy.

The mammalian X and Y chromosomes are imbalanced. The mammalian Y chromosome has lost over 640 genes compared with the X chromosome, and this extensive loss of gene copies has created a gene dosage problem [1]. One solution to counteract the loss of gene copies on the Y chromosome is to adjust the expression level of the copy of the gene that remains on the X chromosome; this ‘dosage compensation’ is often achieved through mechanisms that double gene expression or inactivate the existing copy of the gene on the X chromosome, or by more complicated gene-by-gene adjustments to maintain genome balance [2]. The existence of dosage compensation systems across phylogenetically diverse species illustrates the importance of correcting the imbalance created by the evolution of mature sex chromosomes [3, 4].

Instead of manipulating expression levels, an alternative way to compensate for gene loss is to move genes from the degraded Y chromosome to other, non-sex chromosome locations in the genome. Moving genes away from the sex chromosome would counteract potential deleterious effects of gene dosage imbalance. One remarkable example is found in the Ryukyu spiny rat, which has lost its entire Y chromosome, thus resulting in a significant gene dosage problem. However, genes found on the Y chromosomes of other rodents have not been lost in the Ryukyu spiny rat, and instead they are found as retrotransposed genes on the autosomes [5, 6]. Retrotransposition can therefore act to compensate genes that were lost when the Y chromosome of the Ryukyu spiny rat disappeared.

Until recently, the complete loss of the Y chromosome coupled with evidence that retrotransposition acts to restore lost genes was thought to be unique to this exceptional Japanese rodent. However, new findings presented by Jennifer Hughes and colleagues [7] in *Genome Biology* change this view by revealing that gene transposition is a more widespread phenomenon for rescuing gene dosage from the ravages of sex chromosome evolution.

## Gene transposition from sex chromosomes is evolutionarily widespread in mammals

Few genes remain on mammalian Y chromosomes, making it difficult to study the history of the Y chromosome. However, in their paper [7], Jennifer Hughes and colleagues analyzed and compared genome sequence data for eight mammalian species (human, gorilla, chimpanzee, rhesus macaque, marmoset, mouse, rat and cattle) to identify seven genes (*AMELY*, *EIF1AY*, *EIF2S3Y*, *KDM5D*, *RPS4Y*, *UBA1Y* and *USP9Y*) that were single copy on the Y chromosome and lost recently in several mammalian lineages but maintained on the Y chromosome in others. Of these seven genes, four (*EIF1AY*, *EIF2S3Y*, *RPS4Y* and *USP9Y*) were lost on the Y chromosome in specific lineages but were found to have transposed to autosomes. While the number of sex-linked genes found to have undergone transposition to the autosomes is small, the four genes identified demonstrate that gene transposition is a much more widespread mechanism in mammals for dealing with gene loss on the Y chromosome, rather than an exceptional case confined to a Y-less rodent.

## Evidence of functionality and transposition outcomes

A potential problem with transposition as a mechanism to maintain function is the possible creation of nonfunctional pseudogenes. Retrotransposition, whereby a processed mRNA is inserted into the genome, is common in mammalian genomes and often generates nonfunctional pseudogenes [8]. To evaluate the functionality of the four transposed genes, Jennifer Hughes and colleagues examined the sequence of the autosomal copy to search for intact open reading frames (ORFs) and used RNA-Seq data from many tissues to examine evidence of expression [7].

There are diverse functional outcomes for the four genes among the species of mammals examined. For *EIFA* (eukaryotic translation initiation factor 1), a nonfunctional retrotransposed pseudogene was found in cattle, but a functional retrotranposed copy of *EIFA* was found in rat and mouse. In the latter two species, the retrocopy was expressed at levels comparable to or even higher than the X-linked copy in many tissues; however, in mouse there were exceptionally high levels of expression in the testis. For *EIF2S3* (eukaryotic translation initiation factor 2 subunit 3), autosomal retrotransposed copies are known to occur in humans, but Jennifer Hughes and colleagues report that the retrogene exists in all primates they studied: human, chimp, gorilla, orangutan, rhesus, baboon, marmoset and squirrel monkey. The genomic location of the *EIF2S3* retrogenes varies among primate species and they are found in three different locations, suggesting that *EIF2S3* retrogenes evolved at least three times. Expression of the retrocopy was enriched in the testis for Old World monkeys, but was expressed in many tissues in New World monkeys. In cattle, a new retrotranposed copy was found, but the ORF was truncated, indicating that this retrocopy was nonfunctional. For *RPS4* (ribosomal protein S4), functional retrogenes were present in mouse, rat and cow, and there were two *RPS4* retrogenes found in wallaby and opossum. In wallaby, the X-linked genes (*RPS4X*) were rendered nonfunctional by an ORF-disrupting mutation and the only functional copies of *RPS4* in the wallaby are the retrogenes. Finally, *UBA1* (ubiquitin-like modifier activating enzyme 1) is missing from the Y chromosome of most primate species, but Hughes et al. found an autosomal copy of *UBA1* in the marmoset genome. This copy was not the result of retrotransposition, but rather was directly transposed from the Y chromosome to the autosomes. Expression of the transposed copy is testis specific in the marmoset.

While few in number, the four Y-linked genes and their transposition to autosomes illustrate the diversity and phylogenetic breadth at which gene transposition has occurred from the Y chromosome in mammals. In three cases, the mechanism was retrotransposition and in one case there was direct transposition. In all cases, transposition appears to maintain the function of genes that are normally on the Y chromosome, providing a way to restore function, and suggesting that these genes are especially dosage sensitive. Of considerable interest are the high levels of expression of the transposed copy compared with the X-linked copy. The expression analyses of Hughes et al. show that the levels of expression for the transposed gene are comparable and often higher than their X-linked counterpart. Does this higher expression reflect the increase in dosage for transposed genes compared with the original single-copy Y status? Retrotransposed genes will now have three copies in males (two on the autosome and one on the X chromosome) compared with the original single copy Y. What are the levels of expression of the Y-linked copy in lineages where these genes are still Y-linked? Are these genes already expressed at a higher level on the Y before transposition, or does the expression level increase for the transposed copy? Alternatively, does expression decrease on the X-linked copy after transposition to accommodate the increased copy status of the transposed gene relative to its original single-copy Y-linked status? And why do these expression adjustments not create deleterious consequences? These questions raised by the new results of Hughes et al. will be important for understanding how changes in gene dosage during sex chromosome evolution relate to phenotype.

## Abbreviation

ORF: open reading frame
